# Supplementary material for: A sensation for inflation: initial swim bladder inflation in larval zebrafish is mediated by the mechanosensory lateral line
Source: bioRxiv. 2023 Apr 24:2023.01.12.523756. Preprint. [Version 4] doi: 10.1101/2023.01.12.523756 (PMC9882242; doi:10.1101/2023.01.12.523756)
Supplement: Supplement 2 [file NIHPP2023.01.12.523756v4-supplement-2.pdf]

bioRxiv preprint doi: https://doi.org/10.1101/2023.01.12.523756; this version posted April 24, 2023. The copyright holder for this preprint (which was not certified by peer review) is the author/funder, who has granted bioRxiv a license to display the preprint in perpetuity. It is made available under aCC-BY 4.0 International license.

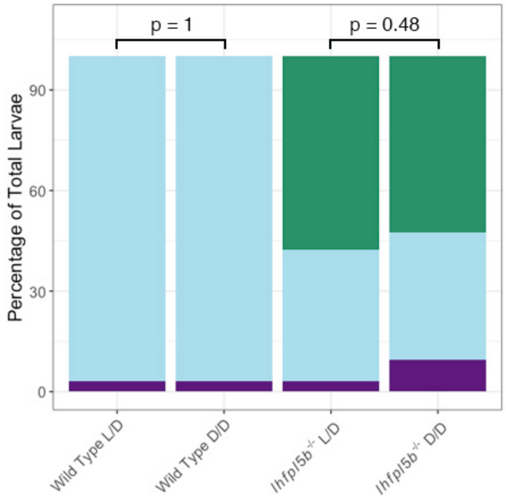

Supplemental Figure 1. Buoyancy proportions of larvae raised in total darkness. Percentage of larvae that exhibit swim bladder under, regular, and over-inflation raised in either a 14:10 light/dark cycle (L/D) or total darkness (D/D). A Chi-Squared Test was used to assess significance. Both Wild Type L/D and Wild Type D/D over-inflation proportions: $M = 0.0\%$, $SD = 0.0\%$, $n = 32$. *lhfpl5b*$^{-/-}$ L/D over-inflation proportion: $M = 57.5\%$, $SD = 6.6\%$, $n = 30$. *lhfpl5b*$^{-/-}$ D/D over-inflation proportion: $M = 52.4\%$, $SD = 4.1\%$, $n = 30$.

bioRxiv preprint doi: https://doi.org/10.1101/2023.01.12.523756; this version posted April 24, 2023. The copyright holder for this preprint (which was not certified by peer review) is the author/funder, who has granted bioRxiv a license to display the preprint in perpetuity. It is made available under aCC-BY 4.0 International license.

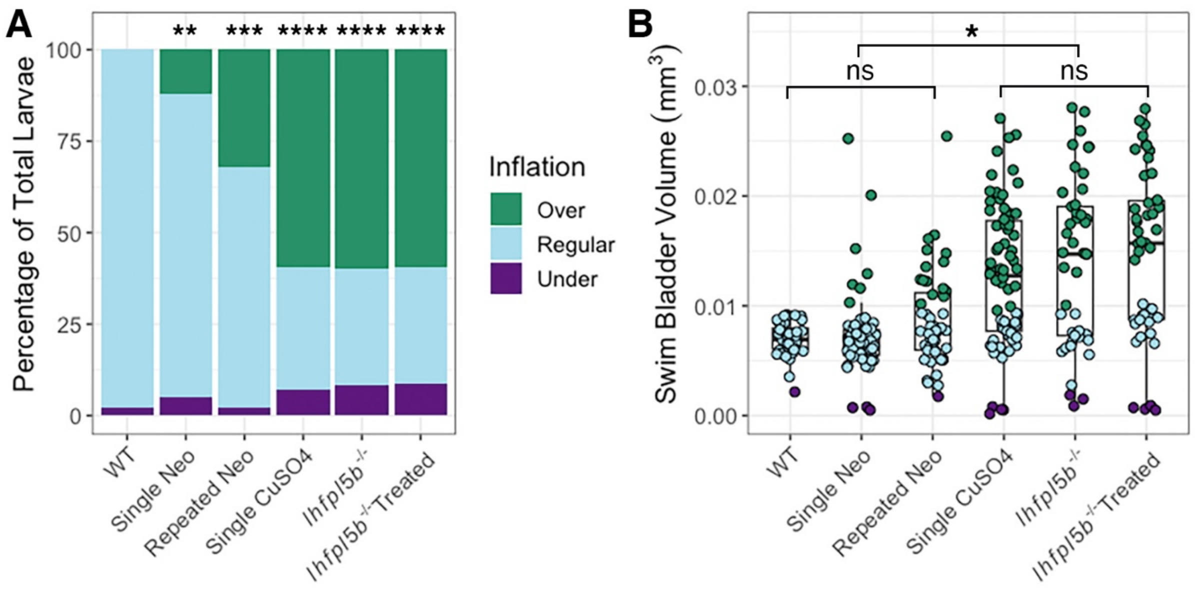

Supplemental Figure 2. Ototoxic ablations of the lateral line. A) Percentage of larvae that exhibit swim bladder under, regular, and over-inflation. A Chi-Squared Test was used to assess significance. B) Swim bladder volume (mm^3) in 5 dpf larvae. A One-Way ANOVA was used to determine significance. **** = $p < 0.0001$, *** = $p < 0.001$, ** = $p < 0.01$, ns = no significance. A full ANOVA table can be found in the supplemental tables.

bioRxiv preprint doi: https://doi.org/10.1101/2023.01.12.523756; this version posted April 24, 2023. The copyright holder for this preprint (which was not certified by peer review) is the author/funder, who has granted bioRxiv a license to display the preprint in perpetuity. It is made available under aCC-BY 4.0 International license.

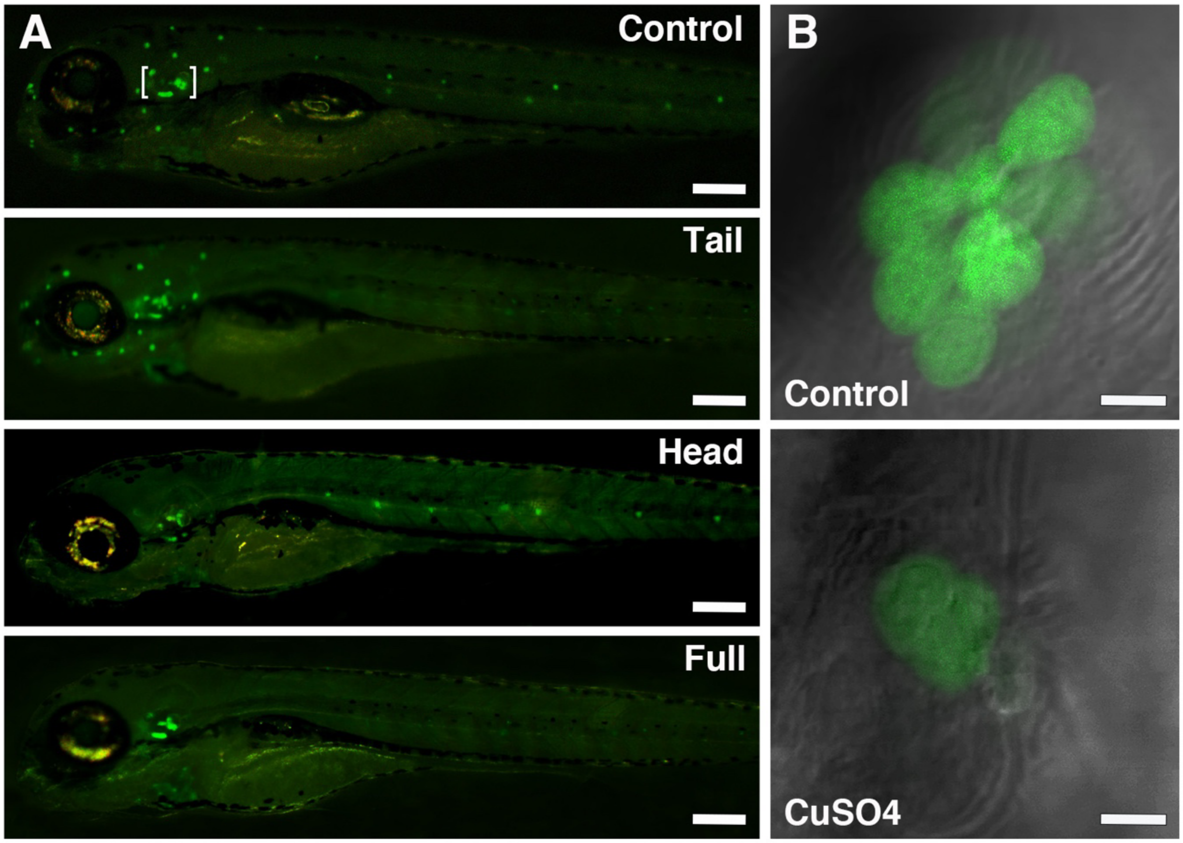

Supplemental Figure 3. Anterior and posterior-specific lateral line ablations with CuSO4. A) Representative images of live *Tg(myo6b:eGFP-pA)vo68* transgenics at 4 dpf, two hours after CuSO4 treatment. This transgene labels hair cells of the lateral line and inner ear (indicated by white brackets on the Control image), and treatment only affects lateral line hair cells so inner ear expression remains post-treatment. B) Representative images from untreated Control and CuSO4-treated L1 neuromasts.

bioRxiv preprint doi: https://doi.org/10.1101/2023.01.12.523756; this version posted April 24, 2023. The copyright holder for this preprint (which was not certified by peer review) is the author/funder, who has granted bioRxiv a license to display the preprint in perpetuity. It is made available under aCC-BY 4.0 International license.

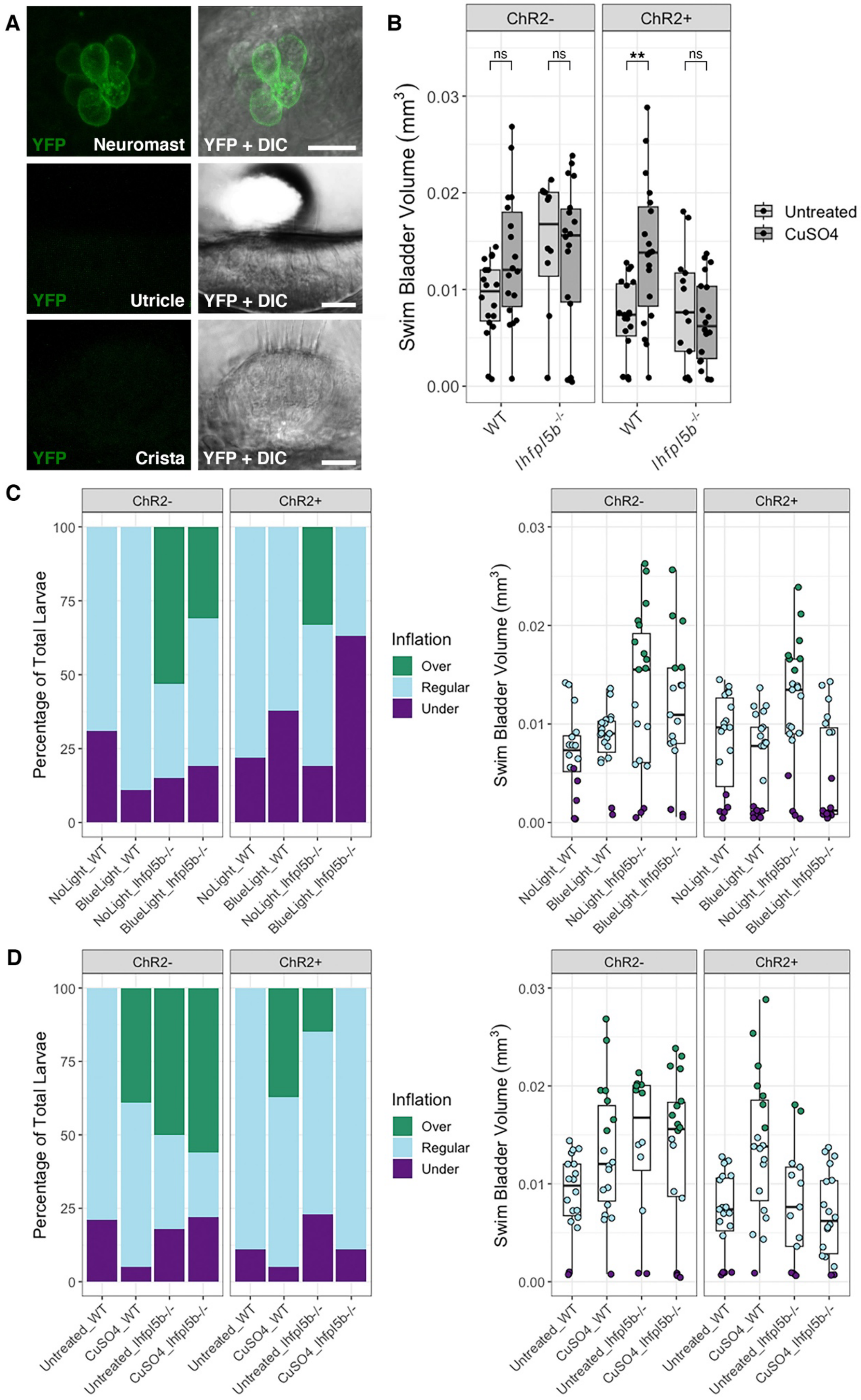

bioRxiv preprint doi: https://doi.org/10.1101/2023.01.12.523756; this version posted April 24, 2023. The copyright holder for this preprint (which was not certified by peer review) is the author/funder, who has granted bioRxiv a license to display the preprint in perpetuity. It is made available under aCC-BY 4.0 International license.

Supplemental Figure 4: Channelrhodopsin-2 (ChR2) expression in *Tg(lhfpl5b2028:ChR2-EYFP-pA)* transgenics. A) Images of ChR2-YFP in hair cells in a 2 dpf neuromast, utricle, and crista where fluorescence expression is only seen in the neuromasts of the lateral line. First panel is YFP only (false-colored green) and the second panel is a merge of YFP and DIC. Scale bars = 10 uM. B) Swim bladder volume (mm^3) of 6 dpf larvae from the experimental condition (blue light overhead). A One-Way ANOVA was used to determine significance. ** = $p < 0.00724$, ns = no significance. A full ANOVA table can be found in the supplemental tables. C) Percentages and box plots of experiment in Figure 6 to show inflation categories. D) Percentages and box plots of experiment in Supplemental Figure 4B to show inflation categories.
