# Supplementary material for: A sensation for inflation: initial swim bladder inflation in larval zebrafish is mediated by the mechanosensory lateral line
Source: bioRxiv. 2023 Jan 26:2023.01.12.523756. Preprint. [Version 2] doi: 10.1101/2023.01.12.523756 (PMC9882242; doi:10.1101/2023.01.12.523756)
Supplement: Supplementary file 1 [file NIHPP2023.01.12.523756v2-supplement-2.pdf]

bioRxiv preprint doi: https://doi.org/10.1101/2023.01.12.523756; this version posted January 26, 2023. The copyright holder for this preprint (which was not certified by peer review) is the author/funder, who has granted bioRxiv a license to display the preprint in perpetuity. It is made available under aCC-BY 4.0 International license.

Supplemental Figures

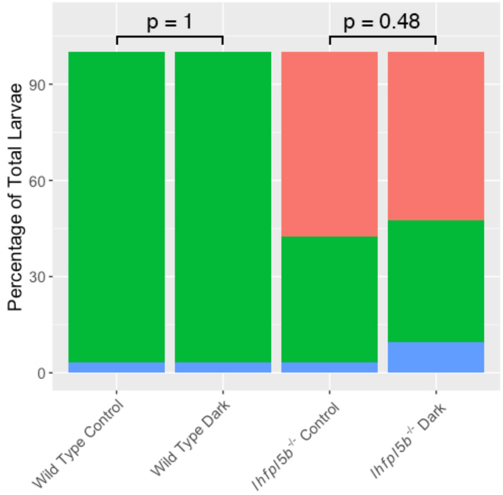

Supplemental Figure 1. Buoyancy status of larvae raised in total darkness. Percentage of larvae that exhibit swim bladder under, regular, and over-inflation raised in either a 14:10 light/dark cycle (Control) or total darkness (0:24 light/dark cycle). A Chi-Squared Test was used to assess significance. Both Wild Type Control and Wild Type Dark over-inflation proportions: $M = 0.0\%$, $SD = 0.0\%$, $n = 32$. *lhfpl5b*$^{-/-}$ Control over-inflation proportion: $M = 57.5\%$, $SD = 6.6\%$, $n = 30$. *lhfpl5b*$^{-/-}$ Dark over-inflation proportion: $M = 52.4\%$, $SD = 4.1\%$, $n = 30$.

bioRxiv preprint doi: https://doi.org/10.1101/2023.01.12.523756; this version posted January 26, 2023. The copyright holder for this preprint (which was not certified by peer review) is the author/funder, who has granted bioRxiv a license to display the preprint in perpetuity. It is made available under aCC-BY 4.0 International license.

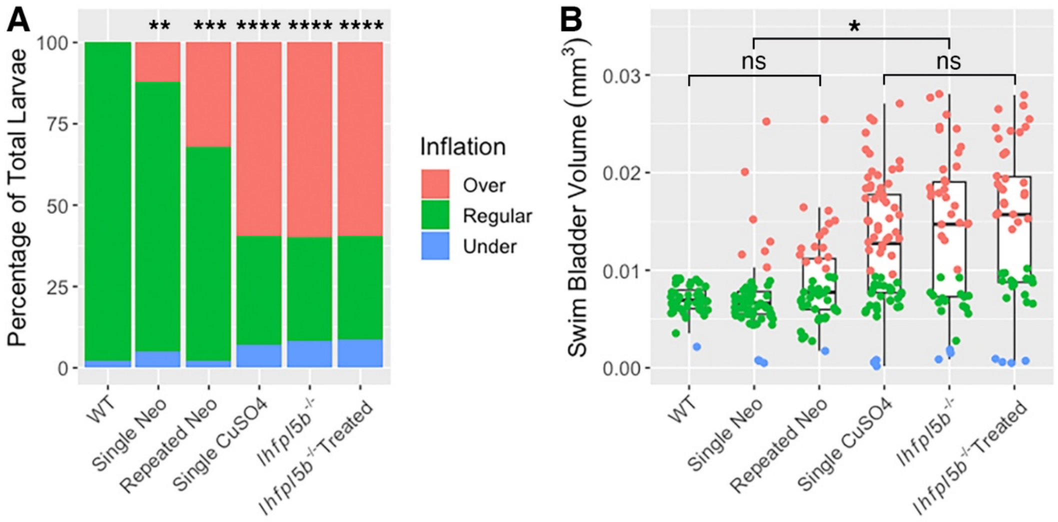

Supplemental Figure 2. Chemical ablations of lateral line hair cells phenocopies the hyperinflation phenotype of *lhfpl5b* mutants. A) Percentage of larvae treated with either neomycin or CuSO4 that exhibit swim bladder under, regular, and over-inflation. A Chi-Squared Test was used to assess significance. B) Swim bladder volume (mm^3) in 5 dpf larvae treated with either neomycin or CuSO4. A One-Way ANOVA was used to determine significance. **** = $p < 0.0001$, *** = $p < 0.001$, ** = $p < 0.01$, * = $p < 0.05$, ns = no significance. A full ANOVA table can be found in the supplemental tables.

bioRxiv preprint doi: https://doi.org/10.1101/2023.01.12.523756; this version posted January 26, 2023. The copyright holder for this preprint (which was not certified by peer review) is the author/funder, who has granted bioRxiv a license to display the preprint in perpetuity. It is made available under aCC-BY 4.0 International license.

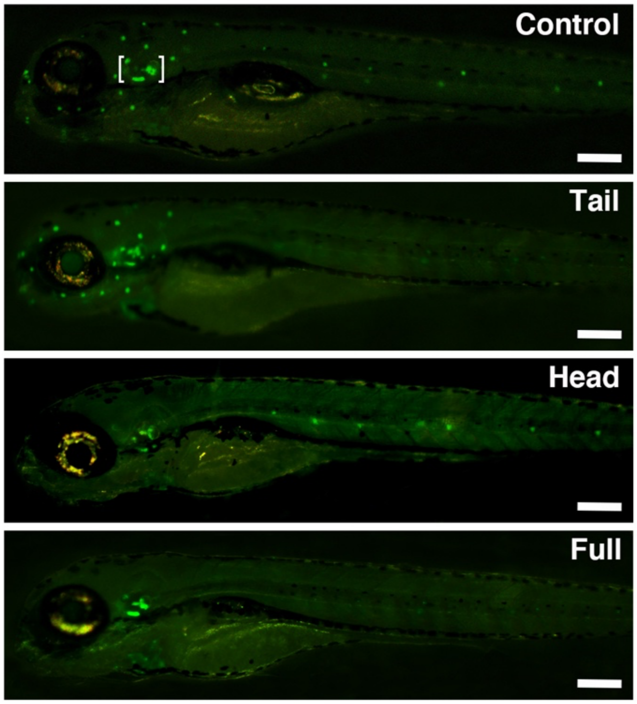

Supplemental Figure 3. Head and trunk-specific lateral line ablations with CuSO4. Representative images of live *Tg(myo6b:EGFP-PA)vo68* transgenics at 4 dpf, two hours after CuSO4 treatment. This transgene labels hair cells of the lateral line and inner ear (indicated by white brackets on the Control image). CuSO4 treatment ablates lateral line hair cells only, leaving inner ear hair cells intact.

bioRxiv preprint doi: https://doi.org/10.1101/2023.01.12.523756; this version posted January 26, 2023. The copyright holder for this preprint (which was not certified by peer review) is the author/funder, who has granted bioRxiv a license to display the preprint in perpetuity. It is made available under aCC-BY 4.0 International license.

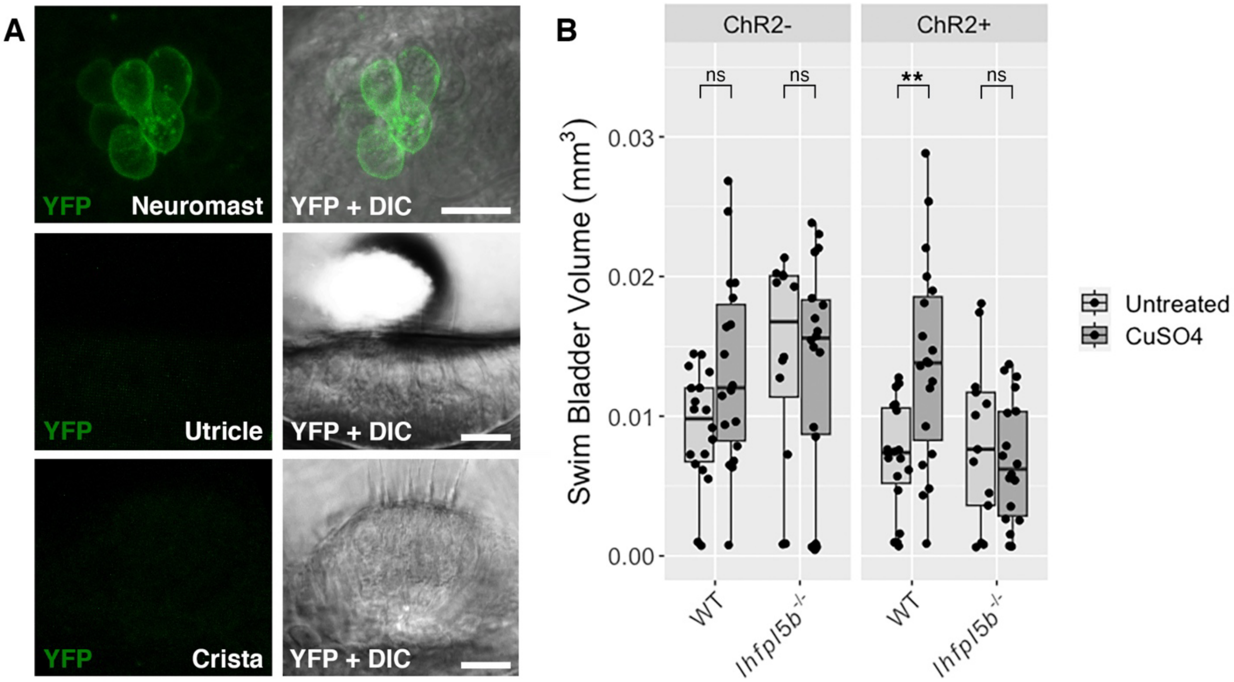

Supplemental Figure 4: A) Representative images of Channelrhodopsin-2 (ChR2) expression in neuromast, utricle, and crista hair cells of *Tg(lhfpl5b2028:ChR2-EYFP-PA)* transgenics at 2 dpf. ChR2-YFP expression is detectable only in the lateral line neuromasts, indicating specificity of the *lhfpl5b* regulatory region used to drive ChR2 expression. First panel is YFP only (false-colored green) and the second panel is a merge of YFP and DIC. Scale bars = 10 uM. B) Swim bladder volume (mm^3) of 6 dpf wild type and *lhfpl5b*$^{-/-}$ larvae, with and without the *ChR2* transgene. Groups of larvae were also treated with CuSO4 to ablate lateral line hair cells. All larvae were stimulated with blue light. The impacts of blue light stimulation on wild type ChR2 transgenics is abolished by chemical ablation of lateral line hair cells. As expected, *lhfpl5b* mutants are unaffected by CuSO4 treatment. A One-Way ANOVA was used to determine significance. ** = $p < 0.00724$, ns = no significance. A full ANOVA table can be found in the supplemental tables.
