# Supplementary material for: A sensation for inflation: initial swim bladder inflation in larval zebrafish is mediated by the mechanosensory lateral line
Source: bioRxiv. 2023 Jan 26:2023.01.12.523756. Preprint. [Version 2] doi: 10.1101/2023.01.12.523756 (PMC9882242; doi:10.1101/2023.01.12.523756)
Supplement: Supplementary file 2 [file media-1.pdf]

Venuto et al. 2023 ANOVA tables

Figure 2 - Blocking Surface Access - ANOVA Table (type II tests)

Effect	DFn	DFd	F	p	p<.05	ges
Status	2	210	50.379	1.43e-24	*	0.419

group1	group2	estimate	conf.low	conf.high	p.adj	p.adj.signif
Blocked MUT	Blocked WT	0.00325	-0.0183	0.0248	0.98	ns
Blocked MUT	Open MUT	0.0831	0.0624	0.104	0	****
Blocked WT	Open MUT	0.0798	0.0599	0.0997	0	****

Figure 3 - Transgenic Rescue - ANOVA Table (type II tests)

Effect	DFn	DFd	F	p	p<.05	ges
Status	2	89	21.699	2.11e- 8	*	0.328

group1	group2	estimate	conf.low	conf.high	p.adj	p.adj.signif
Tg MUT	Tg WT	0.000173	-0.00194	0.00228	0.979	ns
Non-Tg MUT	Tg MUT	0.00567	-0.00825	-0.00309	3.2e- 6	****
Non-Tg MUT	Tg WT	0.0055	-0.00756	-0.00344	2.6e- 8	****

Figure 4 – Region Specific Lateral Line Ablations - ANOVA Table (type II tests)

Effect	DFn	DFd	F	p	p<.05	ges
Status	3	142	7.442	1.15e- 3	*	0.136

group1	group2	estimate	conf.low	conf.high	p.adj	p.adj.signif
Head	Control	-0.00398	-0.00778	-0.000175	0.0365	*
Head	Full	0.000479	-0.00327	0.00423	0.987	ns
Head	Tail	-0.00524	-0.0091.	-0.00138	0.0031	**
Control	Full	0.00446	0.000655	0.00826	0.0145	*
Control	Tail	-0.00126	-0.00517	0.00264	0.835	ns
Full	Tail	-0.00572	-0.00958	-0.00186	9.9e- 4	***

Figure 5 – Surface Oil - ANOVA Table (type II tests)

Effect	DFn	DFd	F	p	p<.05	ges
Status	3	108	8.428	4.4e- 5	*	0.19

group1	group2	estimate	conf.low	conf.high	p.adj	p.adj.signif
MUT Control	MUT Oil	-0.00398	-0.00477	0.000914	0.133	ns
MUT Control	WT Control	0.000479	-0.00763	-0.00194	0.0037	**
MUT Control	WT Oil	-0.00524	0.00154	0.00705	0.886	ns
MUT Oil	WT Control	0.00446	-0.00286	0.0026	0.0523	ns
MUT Oil	WT Oil	-0.00126	0.0063	0.0116	0.0125	*
WT Control	WT Oil	-0.00572	0.00916	0.0144	9.1e- 5	****

Figure 6 – Channelrhodopsin-2 - ANOVA Table (type II tests)

Effect	DFn	DFd	F	p	p<.05	ges
Status	7	142	4.734	8.4e -5	*	0.189

group1	group2	estimate	conf.low	conf.high	p.adj	p.adj.signif
Con.Neg.Mut.	Con.Neg.WT.	-0.00582	-0.0113	-0.000303	0.035	*
Con.Neg.Mut.	Exp.Neg.Mut.	0.00145	-0.00688	0.00397	0.895	ns
Con.Neg.Mut.	Exp.Neg.WT.	-0.00472	-0.00999	0.000555	0.0955	ns
Con.Neg.WT.	Exp.Neg.Mut.	0.00437	-0.00130	0.0100	0.187	ns
Con.Neg.WT.	Exp.Neg.WT.	0.00110	-0.00442	0.00662	0.953	ns
Exp.Neg.Mut.	Exp.Neg.WT.	-0.00327	-0.00869	0.00216	0.394	ns
Con.Pos.Mut.	Con.Pos.WT.	-0.00298	-0.00770	0.00175	0.354	ns
Con.Pos.Mut.	Exp.Pos.Mut.	-0.00687	-0.0115	-0.00221	0.00127	**
Con.Pos.Mut.	Exp.Pos.WT.	-0.00563	-0.0102	-0.00109	0.00893	**
Con.Pos.WT.	Exp.Pos.Mut.	-0.00389	-0.00873	0.000946	0.158	ns
Con.Pos.WT.	Exp.Pos.WT.	-0.00265.	-0.00737	0.00207	0.458	ns
Exp.Pos.Mut.	Exp.Pos.WT.	0.00124	-0.00342	0.00589	0.897	ns

Figure 7 – Behavior Surface Visits - ANOVA Table (type II tests)

Effect	DFn	DFd	F	p	p<.05	ges
Status	2	18	19.513	3.11e- 5	*	0.684

group1	group2	estimate	conf.low	conf.high	p.adj	p.adj.signif
MUT Over	MUT Regular	-55.4	-79.5	-31.3	4.2e- 5	****
MUT Over	WT Regular	-45.3	-69.4	-21.2	4.1e- 4	***
MUT Regular	WT Regular	10.1	-14.0	34.3	0.542	ns

Figure 7 – Behavior Surface Time - ANOVA Table (type II tests)

Effect	DFn	DFd	F	p	p<.05	ges
Status	2	18	24.063	8.21e- 6	*	0.728

group1	group2	estimate	conf.low	conf.high	p.adj	p.adj.signif
MUT Over	MUT Regular	-96.7	-136.0	-57.5	1.7e- 5	****
MUT Over	WT Regular	-87.1	-126.0	-47.9	6.3e- 5	****
MUT Regular	WT Regular	9.57	-29.6	48.8	0.81	ns

Figure 7 – Behavior Time to First Visit - ANOVA Table (type II tests)

Effect	DFn	DFd	F	p	p<.05	ges
Status	2	18	1.295	0.298	-	0.126

group1	group2	estimate	conf.low	conf.high	p.adj	p.adj.signif
MUT Over	MUT Regular	32.1	-23.5	87.8	0.326	ns
MUT Over	WT Regular	3.86	-51.8	59.5	0.983.	ns
MUT Regular	WT Regular	-28.3	-83.9	27.4	0.415	ns

Supplemental Figure 2 – Lateral Line Ablations - ANOVA Table (type II tests)

Effect	DFn	DFd	F	p	p<.05	ges
Status	5	306	18.314	6.63e- 16	*	0.23

group1	group2	estimate	conf.low	conf.high	p.adj	p.adj.signif
Mutant	Mutant Treat	0.00089	-0.00253	0.00431	0.976	ns
Mutant	Repeat Neo	-0.00509	-0.00849	-0.00168	3.51e- 4	*
Mutant	Single CuSO4	-0.00109	-0.00419	0.00201	0.915	ns
Mutant	Single Neo	-0.00662	-0.00986	-0.00337	1.88e- 7	****
Mutant	Wild Type	-0.00682	-0.0103	-0.00336	5.4e- 7	****
Mutant Treat	Repeat Neo	-0.00598	-0.00936	-0.00259	1.06e- 5	****
Mutant Treat	Single CuSO4	-0.00198	-0.00506	0.0011	0.441	ns
Mutant Treat	Single Neo	-0.00751	-00107	-0.00428	1.71 e- 9	****
Mutant Treat	Wild Type	-0.00771	-0.0112	-0.00427	7.61 e- 9	****
Repeat Neo	Single CuSO4	0.00400	0.000936	0.00706	2.94e- 3	**
Repeat Neo	Single Neo	-0.00153	-0.00473	0.00168	0.746	ns
Repeat Neo	Wild Type	-0.00174	-0.00516	0.00169	0.694	ns
Single CuSO4	Single Neo	-0.00553	-0.00841	-0.00265	1.18e- 6	****
Single CuSO4	Wild Type	-0.00573	-0.00886	-0.00261	3.98e- 6	****
Single Neo	Wild Type	-0.000206	-0.00347	0.00306	1.0	ns

Supplemental Figure 4 – Channelrhodopsin-2 Ablations - ANOVA Table (type II tests)

Effect	DFn	DFd	F	p	p<.05	ges
Status	7	127	4.678	0.000108	*	0.205

group1	group2	estimate	conf.low	conf.high	p.adj	p.adj.signif
Tr.Neg.MUT.	Tr.Neg.WT.	-0.000109	-0.00605	0.00583	1	ns
Tr.Neg.MUT.	Un.Neg.MUT.	0.000809	-0.00583	0.00745	0.988	ns
Tr.Neg.MUT.	Un.Neg.WT.	-0.00431	-0.0102	0.00163	0.232	ns
Tr.Neg.WT.	Un.Neg.MUT.	0.000918	-0.00572	0.00756	0.983	ns
Tr.Neg.WT.	Un.Neg.WT.	-0.00420	-0.0101	0.00174	0.253	ns
Un.Neg.MUT.	Un.Neg.WT.	-0.00512	-0.0118	0.00152	0.187	ns
Tr.Pos.MUT.	Tr.Pos.WT.	0.00702	0.00220	0.0118	0.00158	**
Tr.Pos.MUT.	Un.Pos.MUT.	0.00128	-0.00406	0.00661	0.922	ns
Tr.Pos.MUT.	Un.Pos.WT.	-0.000246	-0.00457	0.00507	0.999	ns
Tr.Pos.WT.	Un.Pos.MUT.	-0.00574	-0.0110	-0.000466	0.0277	*
Tr.Pos.WT.	Un.Pos.WT.	-0.00677	-0.0115	-0.00202	0.00207	**
Un.Pos.MUT.	Un.Pos.WT.	-0.00103	-0.00631	0.00425	0.955	ns
